# Advanced Recycled Polyethylene Terephthalate Aerogels from Plastic Waste for Acoustic and Thermal Insulation Applications

**DOI:** 10.3390/gels4020043

**Published:** 2018-05-17

**Authors:** Hong Wei Koh, Duyen K. Le, Gek Nian Ng, Xiwen Zhang, Nhan Phan-Thien, Umeyr Kureemun, Hai M. Duong

**Affiliations:** 1 Department of Mechanical Engineering, National University of Singapore, Singapore 117575, Singapore; a0124203@u.nus.edu (H.W.K.); mpelkd@nus.edu.sg (D.K.L.); a0125030@u.nus.edu (G.N.N.); nhan@nus.edu.sg (N.P.-T.); u.k@u.nus.edu (U.K.); 2 Singapore Institute of Manufacturing Technology, A*STAR, Singapore 637662, Singapore; zhangxw@SIMTech.a-star.edu.sg

**Keywords:** aerogels, recycled PET fiber, plastic waste, acoustic insulation, thermal insulation, mechanical property

## Abstract

This work presents for the first time, a simple, practical and scalable approach to fabricating recycled polyethylene terephthalate (rPET) aerogels for thermal and acoustic insulation applications. The rPET aerogels were successfully developed from recycled PET fibers and polyvinyl alcohol (PVA) and glutaraldehyde (GA) cross-linkers using a freeze-drying process. The effects of various PET fiber concentrations (0.5, 1.0 and 2.0 by wt.%), fiber deniers (3D, 7D and 15D) and fiber lengths (32 mm and 64 mm) on the rPET aerogel structures and multi-properties were comprehensively investigated. The developed rPET aerogels showed a highly porous network structure (98.3–99.5%), ultra-low densities (0.007–0.026 g/cm^3^), hydrophobicity with water contact angles of 120.7–149.8°, and high elasticity with low compressive Young’s modulus (1.16–2.87 kPa). They exhibited superior thermal insulation capability with low thermal conductivities of 0.035–0.038 W/m.K, which are highly competitive with recycled cellulose and silica-cellulose aerogels and better than mineral wool and polystyrene. The acoustic absorption results were also found to outperform a commercial acoustic foam absorber across a range of frequencies.

## 1. Introduction

Polyethylene terephthalate (PET) has been popularly used to produce disposable soft drink bottles since the 1980s; more than 700 million pounds of PET were consumed in 1987 and this number has quickly exploded [1]. Almost all plastic wastes are toxic and pose a direct hazard to the environment. They are seen as noxious materials due to their substantial fraction by volume in the waste stream. Due their high resistance to atmospheric and biological agents, plastic waste is considered as non-biodegradable [2]. Most plastic, including PET can take up to hundreds to years to decompose, and usually end up in oceans and landfills.

Approximately 6300 million tons of plastic waste were generated in 2015 but alarmingly, only 9% was recycled and a large proportion (79%) ended up in landfills, rivers and the oceans. If the current production and waste management trends continue, an estimated 12,000 million tons of plastic waste will be in landfills or in the natural environment by 2050 [3]. Plastic waste, known as marine debris in the ocean, can potentially lead to entanglement and ingestion by marine life and effectively kill the marine life through starvation and debilitation [4]. It is estimated that by 2050, there will be more plastic than fish by weight if no significant actions are taken [5]. Landfill-wise, the plastic waste within landfills may not pose a significant risk to groundwater contamination if managed correctly, but such trends can eventually lead to land scarcity as well as wastage of land area which could be allocated for other useful purposes, and potentially destroy the aesthetics of the urban environment.

Global warming is a climate change phenomenon which results in an increase in the Earth’s overall temperature and is attributed to increased greenhouse gas emissions amplifying the greenhouse effect [6]. With extreme weather and temperature fluctuations, good and effective thermal insulation is crucial in maximizing the energy efficiency in buildings. It retards the rate of heat flow by conduction, convection, and radiation, into or out of buildings due to its high thermal resistance [7,8]. With population growth and rising demand for comfort levels and time spent inside buildings, the consumption of heating, ventilation and air-conditioning systems is approximately 50% of the energy use in buildings [8]; this is especially the case in a city state like Singapore. Therefore, the use of high performance superinsulation materials, which are superior to conventional insulation materials can ensure both energy and cost savings [9].

In addition, noise pollution is currently a major environmental causing negative effects on health, which can be manifest in the form of physiological damage or psychological harm through a variety of mechanisms such as loss of hearing, high blood pressure, and increased physiological stress [10,11]. Exposure to high levels of noise can have an effect on neurocognitive function, mood disorders and neurodegenerative disease [12,13]. Several studies have been conducted to reduce noise pollution by using acoustic insulation materials which are considered as natural (cotton, cellulose, hemp, and stone or vegetal wool), and recycled (rubber, synthetic fibers, plastic, and cork) materials [14,15,16]. They generally have good acoustic insulation properties, similar to traditional porous materials, but may cause some problems with respect to health, the environment, flammability and installation. Silica aerogels are also a good option for acoustic insulation with many advantages such as friendly materials, high flame resistance and good sound absorption properties [17,18]. However, their brittleness and high cost hinder their adoption and industrial development.

Here we report the development of an aerogel utilizing the recycled polyethylene terephthalate (rPET) fibers from plastic bottles waste and its possible large scale thermal and acoustic applications, which are sustainable and environment-friendly. Aerogels are lightweight materials with high porosities (70–99.8%), low densities (0.003–0.5 g/cm^3^), and low thermal conductivities (0.005–0.045 W/m.K) [19,20,21,22]. They are a diverse class of amazing materials with advanced properties. Transparent super-insulating silica aerogels exhibit the lowest thermal conductivity of any known solid. Ultra-strong, bendable x-aerogels are the lowest density structural materials. Normally aerogels are brittle and fracture under too much force. Overcoming the characteristic stiffness of the aerogels could open up a whole new range of uses.

Aerogel-enhanced materials are known to have significantly lower thermal conductivity than traditional insulating materials. However, given the lack of long-term experiences with aerogel-enhanced products, the consistency of their superior thermal performance under different aging in laboratory conditions, and under different humidity and temperature conditions is rarely reported. Some studies [23,24] report that the increase in thermal conductivity in pristine conditions is typically below 10% for aging exposure corresponding to 20 years in typical conditions. Despite some aging-driven degradation, the thermal conductivity of aerogel-enhanced materials after aging remains significantly lower than that of non-aged traditional insulating materials [23,24].

Similar environmental-friendly aerogels such as cellulose aerogels and silica–cellulose hybrid aerogels made from paper waste are known for their high performance, low cost and durability [25,26,27]. The eco-aerogels from other environmental wastes such as paper and fabric waste have been used for several applications such as sound and heat insulation, oil spill cleaning and stopping severe liquid leakage [22,25,28]. Simple and cost-effective synthesis methods for manufacturing biocompatible cellulose-based aerogels using recycled cellulose fibers of paper and cotton waste and Kymene cross-linkers have been developed. However, based on our best knowledge, there are no studies of recycling plastic bottle waste into advanced PET aerogels. The usage and effectiveness of the aerogels in thermal and acoustic insulation have also been reported for architectural purposes, piping, industrial heating systems, cold-storage appliances and anechoic chambers [21,29,30,31,32]. To ensure the stability of their thermal and acoustic insulation performance, it is important that the aerogels exhibit strong hydrophobicity to withstand moisture accumulation. The enhancement of their hydrophobic properties can be achieved through coating with methoxytrimethylsilane (MTMS) [26,33]. In this study, rPET aerogels are successfully developed for the first time from rPET fibers using the freeze-drying process. Their morphology, hydrophobicity, thermal conductivity, acoustic absorption and mechanical properties are comprehensively investigated to assess their thermal and acoustic insulation capabilities. The heat and acoustic insulation performance of the rPET aerogels is also compared to commercial products.

## 2. Results and Discussion

### 2.1. Morphologies and Structures of the rPET Aerogels

**Figure 1:**
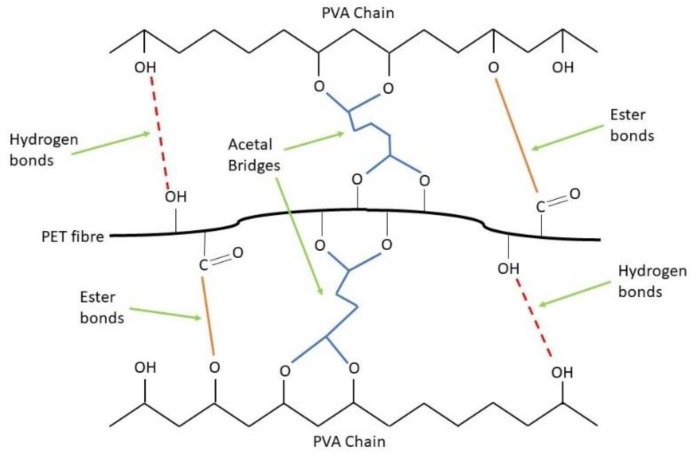
Formation mechanism among the rPET fiber, PVA and GA [35,37].

The rPET aerogels were fabricated through hydrogen and ester bonds formed between polyvinyl alcohol (PVA) and the rPET fibers and acetal bridges from glutaraldehyde (GA) cross-linkers [34,35], as shown in Figure 1. The GA cross-linker with the addition of HCl (controlled at pH ~ 3) can improve the interactions between the rPET fibers and the PVA cross-linker. It also reinforces the rPET-PVA fiber matrix by improving its stability and mechanical properties during the curing process [35,36,37].

**Figure 2:**
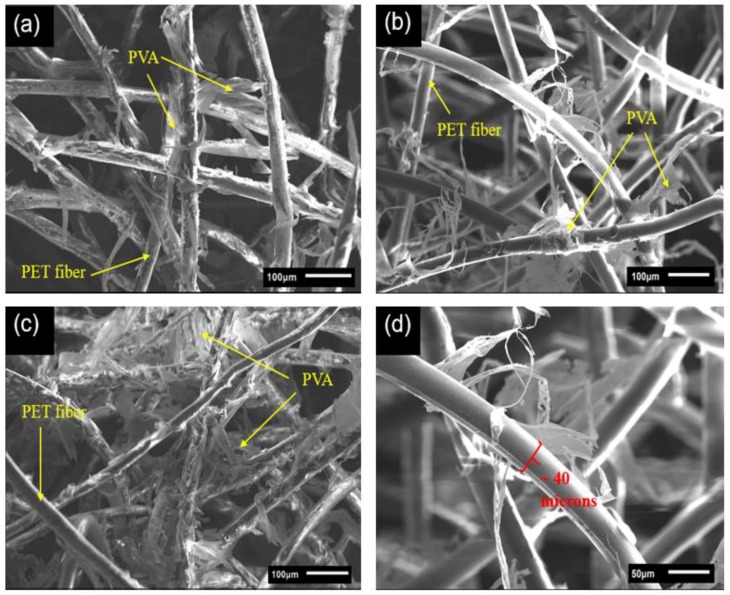
SEM images of the rPET aerogels with the same fiber denier 7D, 64-mm fiber length at different rPET fiber concentrations of (**a**) 0.5 wt.%; (**b**) 1.0 wt.%; and (**c**) 2.0 wt.%; and (**d**) Surface structure of the rPET fibers.

Figure 2 shows the captured SEM images of the rPET aerogels with different rPET fiber concentrations (0.5, 1.0 and 2.0 wt.%). Vastly different from the mesoporous structure (2–50 nm) of the silica–cellulose aerogels [21,26], the rPET aerogels exhibit three-dimensional highly porous network structures with macropores (>50 nm) together with good PVA distribution within the fiber matrix. Comparing Figure 2a–c, it is shown that under the same magnification of 150×, the compactness of the rPET fibers increases when the PET fiber concentration increases from 0.5 to 2.0 wt.%. This results in reduced pore sizes and porosities of the rPET aerogels. Figure 2d shows that the surface structure of the rPET fibers is approximately 40-μm in diameter. The PVA cross-linker can cover the surface of the rPET fibers and cross-link them together.

The rPET aerogels exhibit high porosity levels (98.3–99.5%) and ultra-low densities (0.007–0.026 g/cm^3^) for all samples of the different rPET fiber concentrations, fiber deniers and fiber lengths; their properties are summarized in Table 1. Increasing the content of the rPET fibers in Figure 2 causes an increased number of rPET fibers per unit area, thus leading to the density increase and the porosity decrease of the rPET aerogel. This results in reduced pore sizes within the aerogel structures. In comparing the rPET aerogels having different fiber deniers, different lengths and the same fiber concentration (2.0 wt.%), there is no significant difference between their densities and porosities.

### 2.2. Super-Hydrophobicity Properties of the rPET Aerogels

**Figure 3:**
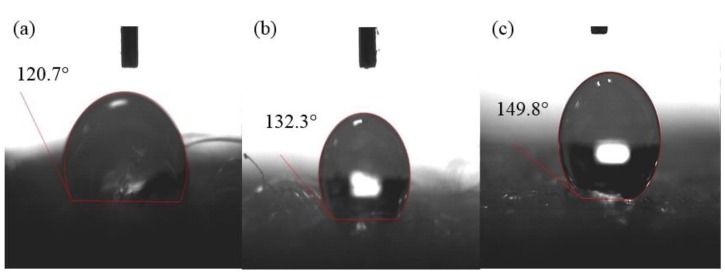
Water contact angles of MTMS-coated rPET aerogels with different rPET fiber concentrations of (**a**) 0.5 wt.% (PPA1 sample); (**b**) 1.0 wt.% (PPA2 sample); and (**c**) 2.0 wt.% (PPA3 sample). The composition details of the aerogel samples are summarized in Table 1.

Figure 3 shows the typical images of the water contact angles on the surface of the rPET aerogels with the various rPET fiber concentrations. They exhibit super-hydrophobic properties with the water contact angles, θ of 120.7–149.8°. The water contact angles increase when the fiber concentrations increase as shown in Figure 3. This can be explained that the aerogel surface is denser due to the addition of more rPET fibers. The super-hydrophobicity of the rPET aerogels is attributed to the stability of the non-polar methyl groups (R–CH_3_) of MTMS replacing their polar hydroxyl groups on the surface [38,39,40]. An explanation for the water contact angle difference is attributed to varying degrees of reaction between the functional alkoxy-silane groups (–Si–O–CH_3_–) of MTMS and the hydroxyl groups on the surface of the rPET aerogels. Our previous studies [21,25,28] also proved that the MTMS-coated aerogels are super-hydrophobic and stable over 6 months under ambient conditions. The super-hydrophobic aerogels can prevent the ambient moisture diffusing in their porous structures. However, the effects of the aerogel surface and the fiber morphologies on the hydrophobic properties should be investigated further.

### 2.3. Acoustic Insulation Properties of the rPET Aerogels

**Figure 4:**
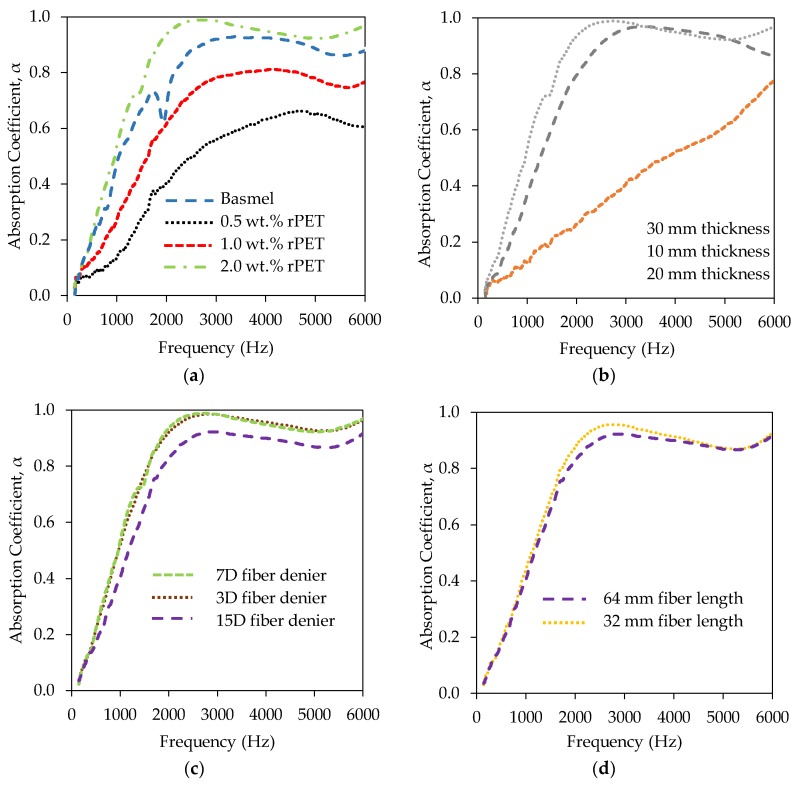
Acoustic performance of the rPET aerogels. (**a**) The rPET aerogels having different fiber concentrations, the same thickness of 30 mm, 7D fiber denier, 64 fiber length compare to the commercial acoustic foam absorber Basmel; (**b**) Effects of different thickness of the rPET aerogels having the same 2.0 wt.% fiber concentration, 7D fiber denier, 64 mm fiber length; (**c**) Effects of various fiber deniers of the rPET aerogels having 2.0 wt.% fiber concentration, 64 fiber length, thickness of 30 mm; (**d**) Effects of fiber length of the rPET aerogels having the same 2.0 wt.% fiber concentration, 15D fiber denier, thickness of 30 mm.

Figure 4a presents the acoustic insulation performance of the rPET aerogels having the different fiber concentrations, the same 30-mm aerogel thickness, the same fiber denier 7D and the same 64-mm fiber length. It is observed that for the same 30-mm aerogel thickness, the absorption coefficient of rPET aerogels increases with an increase in the rPET fiber concentration. An increase in the rPET fiber concentration can increase the number of the rPET fibers per unit area, the fiber-to-fiber contact areas, and tortuosity as well as fiber entanglement [41]. Consequently, there is more energy lost to the propagated acoustic waves due to increasing surface friction [42] and the internal friction caused by a high number of internal reflections [43]. As a result, the level of absorption increases. Among the different fiber concentrations investigated, the 2.0 wt.% rPET aerogels demonstrated the best acoustic absorption results and was about 20–30% better than that of the 1.0 wt.% aerogels across the entire range of frequencies. Compared to a commercial Basmel material with the same thickness and noise reduction coefficient (NRC) of 0.40, the 2.0 wt.% rPET aerogel showed an NRC of 0.45 and performed 20% better at the frequencies of 2000–2500 Hz.

Figure 4b presents the effects of the different thicknesses of rPET aerogels with the same 2.0 wt.% fiber concentration, the same 7D fiber denier and the same 64-mm fiber length on their acoustic insulation performance. With the same 2.0 wt.% rPET concentration, the absorption coefficient, α of the rPET aerogel increases as its thickness increases at the low- to mid-range frequencies (up to 3000 kHz). The 30-mm thickness rPET aerogels exhibit the best acoustic insulation performance and are ~15–20% better than the 20 mm thickness aerogel samples at the lower frequencies. The lower frequency causes the higher wavelength and acoustic waves to be absorbed more when the aerogel thickness increases. The aerogel thickness was also observed to have an insignificant effect on the absorption coefficient at the higher frequencies (>3000 Hz). These findings are consistent to previous reports on biopolymer foams and fiberglass fibers [44,45,46,47]. After reaching its peak at the higher frequencies, there is a slight decrease in the acoustic absorption coefficient of the aerogel samples in Figure 4b. This can be explained by the coincidence dip phenomenon, which can severely limit acoustic absorption ability when the incident acoustic wave is in phase with the reflected wave from the aerogel samples [41,45].

Figure 4c presents the effects of the 3D, 7D and 15D fiber deniers of the rPET aerogels having the same 2.0 wt.% fiber concentration, the same 64-mm fiber length and the same 30-mm average thickness on their acoustic insulation performance. The acoustic absorption coefficients in Figure 4c increase with a decrease in the fiber denier. The 3D- and 7D-denier aerogels perform better than the 15D-denier aerogels. When the aerogel samples have almost identical volume and density, the more rPET fibers can increase the tortuosity and cause higher airflow resistance by frictional viscosity [41,42]. This leads to an increase in absorption. In addition, the finer fibers can displace relatively easier in comparison to the coarser fibers, leading to the faster rate of conversion of acoustic energy to heat [41]. The absorption coefficients of the aerogels with the 3D and 7D fiber deniers are not significantly affected by the small fiber diameters.

Figure 4d shows that the rPET aerogels with a 32-mm fiber length has a slightly better acoustic absorption coefficient than the aerogels with a 64-mm fiber length at the frequencies of 2000–3000 Hz. This is explained that for the same volume and density, the more rPET fibers with a shorter length may lead a more tortuous path and increase the fiber-to-fiber contact areas within the fiber matrix [41,42]. Thus, more energy can be lost through the friction leading to better acoustic absorption.

### 2.4. Thermal Properties of the rPET Aerogels

The thermal conductivities, *K*, of the various rPET aerogel samples having different PET fiber concentrations were determined and are summarized in Table 2. The rPET aerogels exhibit ultra-low thermal conductivities of 0.035–0.038 W/m.K due to their highly porous networks. At ambient temperature and pressure conditions, the trapped air within the highly porous aerogels is a substantial contributor to their low thermal conductivities [48,49]. As shown in Table 2, the thermal conductivities of the rPET aerogels (0.035–0.037 W/m.K) increase with increasing fiber concentrations (0.5–2.0 wt.%) and densities (0.007–0.026 g/cm^3^) and are significantly lower than that of pure rPET fibers (0.15 W/m.K) [50,51,52]. The range of fiber deniers and lengths used in this study has no significant effect on the thermal conductivities of the rPET aerogels.

The thermal conductivities (0.035–0.038 W/m.K) of the rPET aerogels are lower than those of silica aerogels (0.036–0.417 W/m.K) [49], and silica-cellulose aerogels (0.039–0.041 W/m.K) [21]. They are also highly competitive compared to the recycled cellulose aerogels (0.029–0.032 W/m.K) [25], and conventional thermal insulation materials such as polyurethane (0.02–0.04 W/m.K), mineral wool (0.035 W/m.K) and polystyrene (0.034 W/m.K) [53].

**Figure 5:**
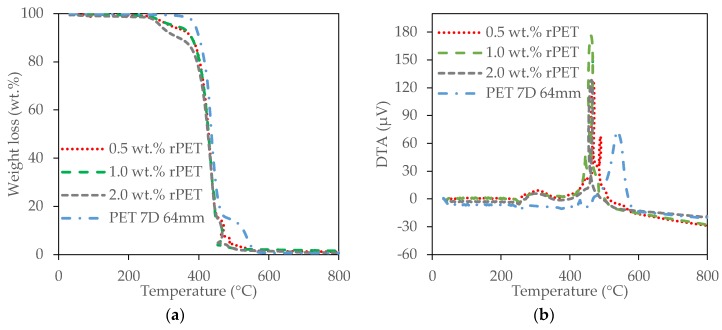
Thermal stability of rPET fiber 7D, 64 mm length and the rPET aerogels with different fiber concentrations. (**a**) TGA; (**b**) DTA.

The thermal stabilities of the rPET aerogels were investigated by thermogravimetric analysis (TGA) and differential thermal analysis (DTA) tests as shown in Figure 5. This indicates that two main weight loss regions are observed with first region seen at approximately 300 °C due to the PVA decomposition on the surface of the rPET aerogels [54]. The second stage weight loss occurs above 420 °C due to the PET fiber decomposition. The higher degradation temperature of the rPET aerogels is 420 °C, which is higher than cellulose aerogels (300 °C) [21] and cellulose-silica aerogels (325 °C) [21,25] and also competitive with the silica aerogels (450 °C) [55].

### 2.5. Mechanical Properties of the rPET Aerogels

**Figure 6:**
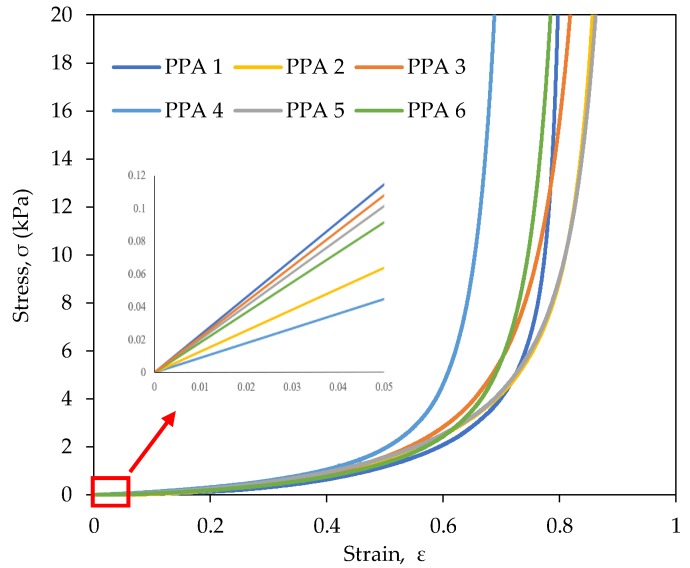
Compressive strain-stress curves of the rPET aerogels having the various rPET fiber concentrations, fiber deniers and fiber lengths. The composition details of the aerogel samples are summarized in Table 1.

The mechanical properties of the rPET aerogels can play a pivotal role in ensuring their durability when they are employed for thermal and acoustic insulation applications. The compressive strain-stress curves and the compressive Young’s modulus, *E* of the rPET aerogels are shown in Figure 6 and Table 2. As shown in Table 2, the compressive Young’s moduli of the rPET aerogels are 1.16–2.87 kPa, much lower than those of the pure silica aerogels (0.1–10.0 MPa) [40] and the silica-cellulose aerogels (86–169 kPa) [21], showing their better durability and reusability than other reported aerogels. The compressive Young’s modulus of the rPET aerogels increases with increasing rPET fiber concentration. Increasing the rPET fiber concentration contributes to the higher rigidity level of the aerogel structures.

The compressive Young’s moduli of the rPET aerogels were observed to decrease slightly with increasing fiber denier (Table 2). With the same volume, decreasing the fiber diameter equates to more rPET fibers per unit area and thus the more compact rPET aerogel structures lead to higher Young’s modulus values. On the other hand, the rPET aerogel sample with a 64-mm fiber length demonstrates a lower Young’s modulus than its counterpart. It is interesting that all the rPET aerogels demonstrated high elastic recovery, returning to approximately 95% of their initial volume after compression.

## 3. Conclusions

Environmentally friendly rPET aerogels were successfully developed from rPET fibers using a freeze-drying process. The effects of the rPET fiber concentrations, the fiber deniers and the fiber lengths on the morphologies and advanced properties of the rPET aerogels were investigated systematically. The developed rPET aerogels showed ultra-low densities (0.007–0.026 g/cm^3^), high porosity (98.3–99.5%), super-hydrophobicity (up to 149.8°), ultra-low thermal conductivities (0.035–0.038 W/m.K) and high elasticities with low compressive Young’s modulus (1.16–2.87 kPa). The rPET aerogels exhibited much better acoustic insulation performance than the commercial acoustic foam absorber. The results demonstrate that rPET aerogels are potential materials for use in a variety of applications such as heat and acoustic insulation, oil spill cleaning, medical devices, personal care products and packaging.

## 4. Materials and Methods

### 4.1. Materials

rPET fibers with deniers of 3D, 7D, and 15D and fiber lengths of 32 mm and 64 mm were purchased from Foshan Rongsheng Furniture Material Co. Ltd. (Guangdong, China). Basmel^®^ was purchased from Acousti-teq Asia PTE. Ltd. (Singapore). Analytical grade methoxytrimethylsilane (MTMS), hydrochloric acid (HCl, 37%), glutaraldehyde solution (GA, 25% in H_2_O), polyvinyl alcohol (PVA) and sodium hydroxide (NaOH) were all purchased from Sigma-Aldrich Pte. Ltd. (Singapore) and used as received without further purification. All the solutions were made with deionized water (DI). 

### 4.2. Fabrication of the rPET Aerogels

Initially the rPET fibers were treated with aqueous NaOH to produce carboxyl and hydroxyl groups on their surface [36]. The rPET fibers were fully immersed in the aqueous NaOH solution of 4.0 wt.% concentration using a fixed ratio of the weight of rPET fibers to the volume of aqueous NaOH (1 g:100 mL). The resulting mixture was heated in an oven for 1 h at 80 °C to accelerate the hydrolysis process. The rPET fibers were washed thoroughly with DI water to remove all the remaining NaOH before immersing them into the mixture of PVA, GA and DI water. The weight ratio of PET fibers:PVA:GA was fixed at 10:1:0.02. The pH of the reaction media was controlled at 3 by HCl (37%) to accelerate the cross-linking reaction. The resulting mixture was sonicated for 30 min at 220–230 W (UIP2000hdT, Hielscher–Ultrasound Technology, Teltow, Germany) for homogenization and removal of bubbles. The cross-linking reaction was carried out in the oven for 3 h at 80 °C and then placed into a freezer for 6–8 h until the sample was frozen. The frozen sample was placed into the freeze dryer (ScanVac CoolSafe freeze dryer, Labogene, Denmark) for 48 h to remove all the solvent and produce the rPET aerogel. The rPET aerogels were fabricated from various fiber concentrations (0.5, 1.0 and 2.0 wt.%), fiber deniers (3D, 7D and 15D) and fiber lengths (32 mm and 64 mm).

The fabricated rPET aerogels were hydrophilic. Surface modification by coating MTMS on their highly porous network structures made them super-hydrophobic. The rPET aerogel samples, together with MTMS contained in a small open glass vial were placed into a sizable container and tightly sealed. The container was then heated in the oven for 24 h at 70 °C for the silanization reaction [22] to obtain super-hydrophobic rPET aerogels.

### 4.3. Characterizations of the rPET Aerogels

Morphologies of the rPET aerogel samples were investigated using a scanning electron microscopy (SEM, JSM-6010PLUS/LV, JEOL Pte. Ltd., Tokyo, Japan). The samples were coated with an approx. 10-nm thin layer of gold for 90 s each by sputtering (Cressington Scientific Instruments Ltd., Watford, UK) prior to SEM to achieve optimal performance through improving its conductivity, with the coating procedure running at a current of 20 mA and voltage of 2.5 kV.

Densities of the rPET aerogels were determined via measurements of the weight and volume of the aerogel samples using a micro weighing scale and Vernier calipers. For the porosity calculation, the skeletal density of the aerogels being equal to the skeletal density of the fibers was assumed in this work, unless the assumption change was stated elsewhere. Porosities, *φ* in a percentage unit, were the measure of void spaces in the aerogel samples and commonly expressed as a percentage of the total volume. The porosities of the rPET aerogels were determined using the densities of the aerogels, *ρ_a_* and the densities of the PET fibers, *ρ_f_* = 1.37 g/cm^3^ [56] as below:

(1) $$\phi =100\times \left(1-\;\frac{\rho_{a}}{\rho_{f}}\right)$$

After undergoing MTMS treatment, the aerogel samples were then taken for a water contact angle test to assess their wettability. The water contact angle tests were conducted via a video contact angle (VCA) goniometer (VCA Optima, AST Products Inc., Billerica, MA, USA) with a built-in software to dispense a desired amount of water droplet (0.5 µL) from a motorized syringe onto the surface of the tested aerogel sample. Visualization of the test was displayed simultaneously on the monitor screen whereby the contact angle could be obtained. Measurements were then repeated at different positions of the aerogel samples to obtain an average value.

Compression testing was carried out using an Instron 5500 micrometer (Instron, Norwood, MA, USA) to investigate the compressive Young’s moduli of the rPET aerogels in which the test samples were under a loadcell of 1000 N at a loading rate of 1.0 mm/min.

C-Therm TCi Thermal Conductivity Analyzer (C-Therm Technologies, Fredericton, NB, Canada) was utilized to provide highly accurate thermal characterization of the aerogels’ thermal conductivities. This system conducted the test using the modified transient plane source method under ambient conditions [57]. A total of three measurements were taken for each aerogel sample and an average reading was obtained.

Thermal stability of the rPET aerogels was evaluated by thermogravimetric analysis (TGA) and differential thermal analysis (DTA) to determine the weight loss in relation to temperature with a Shimadzu DTG60H, Tokyo, Japan. The specimen was heated to 800 °C from room temperature at the rate of 10 °C/min in air. During the heating process, the specimen was maintained at 150 °C for 1 h to remove the absorbed water.

Acoustic insulation capabilities of the aerogels were investigated using SW422 and SW477 Impedance Tubes (SW series, BSWA Technology Co., Ltd., Beijing, China) to accurately measure the acoustic absorption coefficients in accordance to ASTM International Standards, ASTM E1050-12 [58] across three different ranges of frequencies of 20–450 Hz, 250–1600 Hz and 1000–6100 Hz. The aerogel samples with the diameter of 100 mm were used for the wider impedance tubes (SW422) for frequencies ranging from 20 to 1600 Hz. The smaller samples with the diameter of 30 mm were used for tests in narrower impedance tubes (SW477) for higher frequencies up to 6100 Hz. The tests were carried out on aerogel samples with different fiber concentrations, deniers and lengths. The best aerogel sample was further compared to a commercial acoustic foam absorber, Basmel, using the noise reduction coefficient (NRC), determined at several octave bands of 250 Hz, 500 Hz, 1000 Hz and 2000 Hz, and rounded off to the nearest multiple of 0.05.

## Figures and Tables

**Table 1:** Densities and porosities of the developed rPET aerogels.

Samples	Fiber Length (mm)	Fiber Denier	Fiber Concentration (wt.%)	Thickness (mm)	Density, *ρ_a_* (g/cm^3^)	Porosity, *φ* (%)
PPA 1	64	7D	0.5	30	0.007 ± 0.001	99.47 ± 0.05
PPA 2	64	7D	1.0	30	0.014 ± 0.001	99.00 ± 0.05
PPA 3	64	7D	2.0	30	0.026 ± 0.001	98.14 ± 0.05
PPA 4	64	3D	2.0	30	0.024 ± 0.001	98.26 ± 0.05
PPA 5	64	15D	2.0	30	0.024 ± 0.001	98.26 ± 0.05
PPA 6	32	15D	2.0	30	0.024 ± 0.001	98.26 ± 0.05
PPA 7	64	7D	2.0	10	0.024 ± 0.001	98.23 ± 0.05
PPA 8	64	7D	2.0	20	0.024 ± 0.001	98.25 ± 0.05

**Table 2:** Thermal conductivity and compressive Young’s modulus properties of the rPET aerogels.

Samples	Fiber Concentration (wt.%)	Density (g/cm^3^)	Thermal Conductivity, *K*_avg_ (W/m.K)	Compressive Young’s Modulus, *E* (kPa)
PPA 1	0.5	0.007 ± 0.001	0.035 ± 0.001	1.16 ± 0.05
PPA 2	1.0	0.014 ± 0.001	0.036 ± 0.001	1.76 ± 0.08
PPA 3	2.0	0.026 ± 0.001	0.037 ± 0.001	2.76 ± 0.16
PPA 4	2.0	0.024 ± 0.001	0.037 ± 0.001	2.87 ± 0.17
PPA 5	2.0	0.024 ± 0.001	0.038 ± 0.001	2.61 ± 0.17
PPA 6	2.0	0.024 ± 0.001	0.037 ± 0.001	2.45 ± 0.21
